# Migrasomes should not be classified as extracellular vesicles

**DOI:** 10.1111/jcmm.18337

**Published:** 2024-05-09

**Authors:** Johann Mar Gudbergsson, Anders Etzerodt

**Affiliations:** ^1^ Department of Biomedicine Aarhus University Aarhus Denmark

## WHAT ARE MIGRASOMES?

Cellular migration requires involvement of several different structures and cellular pathways that allow the cell to sense the surrounding environment and move through it. During migration, retraction fibers (RFs) are left behind the trailing edge of the cell or from retracted cellular protrusions such as lamellipodia or filopodia.^1^ Formation of vesicle‐like structures in RFs has recently been described and named as ‘migrasomes’,^2^ and was shown to form by clustering of tetraspanin‐ and cholesterol microdomains in RFs which ultimately ‘swells’ and becomes a migrasome.^3^ Finally, migrasomes have been proposed to degrade over time and release their content to the extracellular space for intercellular communication purposes.^2^ The main marker used to identify migrasomes has been TSPAN4 and the characterization has mostly been done in TSPAN4‐overexpressing cells. However, the migrasome structures themselves are not new and identical structures have been identified three decades ago and many times since, although, their name has not been coined until recently.^4^, ^5^, ^6^, ^7^

Since the first characterization of migrasomes was published in 2015 only few migrasome publications have studied its function in vivo. In a study by Jiang et al,^8^ the disintegration of migrasomes and their subsequent release of content to the extracellular space was suggested to serve as an essential means of cell communication during zebrafish gastrulation where the migrasome content served as chemoattractant in organ morphogenesis. Similarly, the same research group showed that migrasomes from monocytes play a vital role in capillary formation in the chorioallantoic membrane of chick embryos by releasing CXCL12 and VEGFA over time, assumingly due to migrasome rupture or leakage.^9^ A study by Jiao et al^10^ recently showed that damaged mitochondria were deposited in migrasomes in order to uphold the general mitochondrial integrity of the cell in a model of mild mitochondrial stress. Here the authors propose that a high energy burden leads to the accumulation of ROS and subsequent mitochondrial stress. As a response to this, affected cells rapidly dispose of damaged mitochondria in migrasome.^10^ In other words, the migrasomes were used as quick and energy‐efficient alternative to cellular garbage disposal. In support of this, Schmidt‐Pogoda et al^11^ saw neuronal fragments co‐localized in migrasomes from microglia during ischemic stroke, which could suggest that microglia use migrasomes as waste bins when cleaning up after neuronal death.

## MIGRASOMES SHOULD NOT BE CLASSIFIED AS EXTRACELLULAR VESICLES

After the discovery of migrasomes, several publications have referred to migrasomes as EVs^8^, ^12^, ^13^, ^14^, ^15^, ^16^, ^17^, ^18^, ^19^, ^20^ or compared them directly to EVs.^21^, ^22^, ^23^, ^24^ The migrasome discovery paper by Ma et al^2^ describes the specific disintegration of RFs resulting in migrasome release to be key for intercellular communication in migrating cells, thus generating a basis for comparison with EVs. However, as their own electron microscopy data shows, the migrasomes are not released from the RFs but still share plasma membrane with joined RFs,^2^ which was recently also confirmed by Zhang et al.^9^ For some of the in‐depth characterization by electron microscopy, density gradient‐based cell fractionation was used to enrich for the migrasomes,^2^ which could potentially allow for co‐enrichment of other membrane protrusion structures such as different filopodia extensions.^1^, ^25^ Dynamic filopodia (referred to as a microspike in 1963^26^) are not attached to a substrate, are generally shorter than RFs, and some of these contain a vesicle‐like structure at the tip similar to the migrasome in RFs, which might make it difficult to distinguish between the two.^1^ In vitro and in vivo *s*tudies have also shown that content is shuttled from the cell to their RFs to form migrasomes. Thus, RFs must be in direct open‐ended contact with the cell until the whole RF‐migrasome complex is detached from the cell as it moves.^10^, ^11^ The current definition of EVs is ‘particles that are released from cells, are delimited by a lipid bilayer, and cannot replicate on their own’.^20^ Since the migrasome is not delimited by a lipid bilayer but is a part of RFs, migrasomes themselves cannot be classified as EVs. Can the whole RF‐migrasome complex then be classified as an EV? In our opinion, this classification is not appropriate, and with current evidence we suggest that the RF‐migrasome complexes should be referred to as ‘released cell fragments’ as described by Mayer et al.^6^

## CONCLUDING REMARKS

There is little doubt that migrasomes are present both in vitro and in vivo and may function as mediators of intercellular communication in migrating cells or cellular waste bins. From the currently available data, it is also clear that a classification of migrasomes as EVs is not appropriate since they have not been shown to be enclosed vesicles nor to be separated from RFs as vesicular structures. Thus, with current evidence, the migrasome can only be structurally described as an integral part of RFs and we suggest that RF‐migrasome complexes are described as ‘released cell fragments’.

## AUTHOR CONTRIBUTIONS

**Johann Mar Gudbergsson:** Conceptualization (lead); supervision (equal); writing – original draft (lead); writing – review and editing (equal). **Anders Etzerodt:** Conceptualization (supporting); supervision (equal); writing – original draft (supporting); writing – review and editing (equal).

## FUNDING INFORMATION

This letter is supported by grants to AE from the Novo Nordisk Foundation (Project grant in basic bioscience and biomedicine ref #0065510).

## CONFLICT OF INTEREST STATEMENT

The authors declare no conflict of interest.

## Data Availability

No data available.
